# A Case for a Multi‐Professional Approved Clinician Role in Child and Adolescent Mental Health Service Inpatient Units, Crisis, and Liaison

**DOI:** 10.1111/jcap.70031

**Published:** 2025-07-31

**Authors:** Naomi Williams

**Affiliations:** ^1^ Warwick Medical School The University of Warwick Coventry UK

**Keywords:** approved‐clinician, CAMHS, inpatient, multi‐professional, neurodivergent, service delivery

## Abstract

This case supports the rationale for implementing a multi‐professional approved clinician (MPAC) role for neurodivergent individuals within child and adolescent mental health services (CAMHS). An MPAC's core discipline is commonly social work, mental health nursing, and occupational therapy, with additional training, experience, and skills in mental health law, policy, and practice. The MPAC role would complement the existing approved clinician role, usually fulfilled by a psychiatrist. Adding the MPAC role would provide a holistic multidisciplinary approach to mental health provision for children and adolescents up to age 25. Furthermore, the introduction of this role will enhance quality of care, improve service efficiency, and generate financial benefits by optimizing resource utilization within CAMHS.

## Summary

- What Is Known?
  ∘ The multi‐professional approved clinician (MPAC) is usually a qualified social worker, mental health nurse, or occupational therapist with specialized skills, experience, and training in the Mental Health Act and evidence‐based mental health care.
  ∘ Neurodivergent individuals up to age 25 have a high likelihood of needing crisis‐focused treatment.
  ∘ Clinicians lack the knowledge, confidence, and skills to deliver psychotherapy to neurodivergent individuals.
- What Is New?
  ∘ The MPAC model can be implemented into existing structures across CAMHS inpatient, crisis, and liaison to complement the role of the current psychiatrist AC.
  ∘ The MPAC role specializes in the mental health and neuroscience of neurodivergent individuals.
- What Is Significant for Clinical Practice?
  ∘ The MPAC can reduce the gaps in provision for neurodivergent individuals up to age 25, by conducting training, consultation, and specialized treatment and discharge models.
  ∘ The MPAC can spearhead pioneering research and influence policy, practice, and mental health service provision.

## 1 Clinical Rationale

Individuals with neurodevelopmental conditions including autism, ADHD, intellectual/learning disabilities, dyslexia, dyspraxia, and Tourette's disorder. Neurodivergent individuals are more likely than their counterparts to need crisis‐focused treatment for comorbid conditions including anxiety, depression, eating, sleep, and trauma‐related disorders (Cooper et al. 2007). There are currently 2040 individuals with an autism and/or learning disability diagnosis in inpatient units (NHS England 2025). In December 2024, of the 210 young people in inpatient units, 98% had an autism diagnosis (The National Autistic Society 2025). Studies have recognized that those with an autism diagnosis are 50%–60% more likely to meet the diagnostic criteria for an overlapping neurodevelopmental condition such as ADHD, learning disabilities, Tourette's disorder, dyspraxia, or learning difficulties (Lau‐Zhu et al. 2019; Embracing Complexity 2022). Unfortunately, clinicians feel less knowledgeable and confident when delivering psychological therapy to neurodivergent individuals (Gallant et al. 2023). Furthermore, it is expected that the need is much higher due to long wait times for diagnosis and treatment; as such, the current statistics do not reflect the actual need.

Therefore, the need to introduce an MPAC role remains. Unfortunately, studies suggest that the experience that neurodivergent individuals have during their time with CAMHS inpatient, crisis, and liaison is traumatic, and many neurodivergent individuals are discharged feeling worse than when they entered the provision (Roe et al. 2024). Furthermore, the stays for neurodivergent individuals in acute inpatient wards are twice as long compared to their counterparts (Nikolić et al. 2024), with an average stay of 4.8 years. The MPAC role would create a unique combination of clinical and research expertise for CAMHS inpatient, crisis, and liaison provision. The MPAC role would offer a new layer of expert clinicians alongside psychiatrists and existing staff. The MPAC would have specialist knowledge and experience in systemic practice, early psychosis, children in care, trauma‐informed care, risk management, complex presentations, and knowledge of a wide range of legal and safeguarding frameworks. Core clinical specialities include neurodevelopmental conditions and co‐occurring mental health conditions. The MPAC role has been piloted sporadically across the UK; however, with meaningful investment, the role has the potential to significantly contribute to mental health service provision (Oates et al. 2021). Unfortunately, the evidence of the benefits of the role in CAMHS inpatient, crisis, and liaison is limited as there is no clear framework to properly integrate the role into existing infrastructures. Budgets for mental health service provision are also crucial for key decision makers, making the financial case for the MPAC role within CAMHS inpatient, crisis, and liaison compelling, as it offers numerous potential cost‐saving benefits. This business case will allow the reader to consider the role of an MPAC in CAMHS inpatient, crisis, and liaison alongside a robust implementation plan.

### 1.1 Enhance Patient Care

The MPAC can use their expertise in neurodevelopmental conditions, comorbidities, and evidence‐based creative therapies such as play, Lego, art, music, drama, and outdoor therapies to provide robust treatment recommendations. This holistic approach will meet the needs of a diverse population where there are currently gaps in knowledge, expertise, and experience for individuals needing crisis‐focused treatment. As a result, there will be tailored, person‐centered interventions which extend beyond traditional psychiatric models of mental health provision in CAMHS inpatient, crisis, and liaison.

### 1.2 Specialized Consultations

The MPAC can provide consultations targeting the factors that reduce the risk of long‐term inpatient stays to improve the likelihood of individuals not reaching a mental state of crisis. Targeted consultations will include sensory integration, sleep, nutrition, and pain management. This comprehensive approach will allow CAMHS inpatient, crisis, and liaison provision to offer an inclusive model of mental health care.

### 1.3 Neuroscience and Evidence‐Based Practice

The MPAC role can be a clinical academic role integrating innovative mental health research and legislation into practice. The adoption of a translational research model, aligned with collaborative efforts to include lived‐experience neurodivergent individuals into clinical practice, will allow the MPAC role to be fit for purpose. In addition, this evidence‐driven approach will allow the continual improvement of clinical interventions, consultations, policies, and procedures. As a result, there will be an improvement in service equity for neurodivergent individuals.

### 1.4 Crisis Management and Risk Mitigation

An MPAC is experienced in managing high‐risk caseloads and can offer strategic clinical insight. The MPAC will complement the AC's role in enhancing the unit's ability to respond to crises and respond to high‐risk patients. Therefore, staff, public, and patient safety should be improved.

### 1.5 Integrated Care Delivery

The MPAC can partner with the AC and other mental health professionals to facilitate care planning and delivery, using relevant legislation such as the Mental Health Act. The benefit of having an MPAC as part of the team from admissions through to discharge is that it will ensure that all aspects of a neurodivergent individual's mental health are considered, such as education, social, and health care. This partnership can lead to effective and coordinated treatment plans to improve patient outcomes.

### 1.6 Global Influence

The MPAC can bring strategic insight and global influence of best practices, ensuring that prospective mental health services that implement the role remain at the forefront of innovation and quality provision.

## 2 Quality Improvement

### 2.1 Reducing Care Gaps

The needs of neurodivergent individuals are often overlooked in aspects of mental health provision, such as issues linked to sensory processing, sleep, and meals. The MPAC can remove systematic barriers to care for this population by designing needs‐led treatment and discharge plans, adhering to “waiting well” protocols. “Waiting well,” coined by the NHS in England (NHS England 2025), refers to an approach originally used to support individuals awaiting surgery. Recently, it has been used for any planned healthcare treatment. It emphasizes optimizing an individual's health and well‐being during the waiting period. Thus, reducing gaps in mental health care and ensuring that all children and adolescents receive quality, person‐centered care.

#### 2.1.1 Measures

- Access metrics: Time from referral to first contact, waiting times between services.
- Transition tracking: Number of patients successfully transitioned between services without gaps.
- Coverage analysis: Geographic and demographic areas served, identification of underrepresented populations.
- Continuity measures: Percentage of patients maintaining the same clinician across service transitions.
- Equity indicators: Access rates by ethnicity, socioeconomic status, and neurodivergent diagnosis.

### 2.2 Evidence‐Based Consultancy and Continuous Professional Development

The MPAC can provide evidence‐based training, workshops, webinars, and consultancy to CAMHS inpatients, crisis, the wider service, and external agencies, ensuring that the latest research and evidence inform clinical practice, specifically in areas where clinicians lack knowledge, confidence, and skills for delivering psychological therapies to neurodivergent individuals. This will enhance mental health service provision and contribute to developing best practices.

#### 2.2.1 Measures

- Consultation logs: Number and type of consultations provided to other staff/services
- Training delivery: Sessions conducted, staff trained, and competency assessments
- Protocol development: Evidence‐based guidelines and pathways created or updated
- Case complexity scoring: Documentation of high‐complexity cases managed
- Supervision records: Quality and frequency of supervision provided to junior staff
- Continuous professional development: Conference attendance, certifications, reflective practice, competency frameworks, and peer learning

### 2.3 Patient and Family Satisfaction

The importance of the patient journey is often referred to in policy writing and discussed when mapping service pathways; it is a key part of building trust and engagement. A patient‐centered approach is crucial to meeting the unique needs of neurodivergent individuals and their caregivers. This will improve patient satisfaction and outcomes for neurodivergent individuals and their caregivers.

#### 2.3.1 Measures

- Experience surveys: Standardized questionnaires
- Coproduction activities: Patient and public involvement and engagement in service development
- Cultural competency: Satisfaction across diverse demographic groups

### 2.4 Reduced Readmissions

The MPAC's preventative approach will allow clinicians to feel support in addressing the underlying issues causing neurodivergent individuals to be in crisis. This robust approach will reduce the rate of readmissions by ensuring patients are discharged with sustainable care plans.

#### 2.4.1 Measures

- Unplanned 30 or 90‐day readmission rates: Compared to baseline and national benchmarks
- Length of stay: Average inpatient duration for MPAC caseload
- Crisis contacts: Frequency of unplanned crisis interventions
- Planned versus unplanned admissions: Ratio tracking
- Community: How long do patients remain successfully in community settings

### 2.5 Data Collection Methods

- Electronic health record dashboards
- Patient‐reported outcome measures
- Staff competency tracking systems
- Regular audit cycles

## 3 Financial Impact

### 3.1 Optimized Resource Utilization

The MPAC role can reduce the reliance on the AC and free up time for consultations within their multidisciplinary expertise, allowing psychiatrists to focus on more complex clinical presentations. As a result, available resources are optimized, and overall costs are reduced. The savings can be reinvested into creating more staffing resources for the service.

### 3.2 Cost Avoidance Through Prevention

By addressing underlying issues for neurodivergent individuals, the MPAC can prevent problems from escalating into severe mental health conditions. Therefore, avoiding the cost associated with acute interventions.

### 3.3 Increased Efficiency

MPACs can work within the multidisciplinary team to reduce duplication and streamline care delivery. The increased efficiency translates into lower operational costs and better use of staff time.

### 3.4 Revenue Generation Potential

The MPAC can contribute to developing specialized programs and services that can be marketed to other NHS trusts, institutions, and private agencies, creating potential revenue income streams.

### 3.5 Reduction in Length of Stay

Hallett et al. (2025) have identified that patients who experience longer periods on the wards are more traumatized by inpatient experience. By providing an all‐inclusive mental health service provision to address holistic needs, it will likely reduce the length of inpatient stays, further contributing to saving costs.

## 4 Implementation Plan

### 4.1 Credentialling Framework

#### 4.1.1 Initial Credentialling

- Minimum qualifications: Registered healthcare professional with 5+ years CAMHS experience
- Core competency assessment: Portfolio‐based evidence against defined standards
- Supervised practice period: 12‐month structured program with senior MPAC or AC
- Final assessment: Multi‐score feedback, case presentations, competency examiniation
- Professional body recognition: NMC, HCPC, BPS, SWE to establish MPAC credentials

#### 4.1.2 Ongoing Credentialling

- Annual revalidation: CPD portfolio, supervision records, patient feedback
- Regular case discussion and competency assessment
- 5‐year re‐credentialling: Comprehensive review including external evaluation

### 4.2 General Discipline Responsibilities

- Complex case management
- Evidence‐based consultation to teams
- Specialist assessment and intervention

### 4.3 Discipline‐Specific Boundaries

- Cannot prescribe medication, unless qualified as a prescriber
- Psychological interventions must be limited to trained modalities with appropriate supervision and governance
- Medical decisions to be made in collaboration with a psychiatrist for complex medical management
- Can lead on safeguarding matters by adhering to statutory reporting procedures

### 4.4 Supervised Onboarding

Trainee MPACs should work under the supervision of existing MPACs and ACs as part of onboarding and training. Time must be allocated for the trainee MPAC to be assigned a senior MPAC who can offer quarterly mentoring over 1 year, which will help them develop their skills and abilities. This 12‐month period is fundamental for building the skills and confidence required to autonomously deliver the responsibilities necessary as an MPAC while making the best use of their existing expertise.

#### 4.4.1 Multi‐Layered Approach

- Primary supervisor: Senior MPAC or consultant‐level clinicians (weekly 1:1)
- Professional supervision: Discipline‐specific supervisor for registration requirements (monthly)
- Peer supervision: MPAC group supervision for complex cases (fortnightly)
- Specialist consultation: Access to psychiatrist, psychologist as needed (ad‐hoc)

#### 4.4.2 Supervision Standards

- Frequency: Minimum 2 h formal supervision weekly in the first year, 1.5 h thereafter
- Documentation: Structured supervision records, action plans, competency tracking
- Escalation pathways: Clear processes for concerns about practice or competence

### 4.5 Training and Development Pathway

#### 4.5.1 Phase 1: Foundation (Months 1–6)

- Core MPAC competencies training
- Shadowing senior MPACs
- Supervised practice with regular feedback
- Assigned lower complexity cases

#### 4.5.2 Phase 2: Consolidation (Months 7–12)

- Increased caseload
- Leading on specific projects
- Providing consultation to junior staff
- Competency assessment milestones

#### 4.5.3 Phase 3: Full Practice (Month 12+)

- Independent MPAC practice
- Mentoring new MPACs
- Service development activities
- Ongoing specialist training

### 4.6 Portfolio Development

During this period, trainee MPACs should receive support to develop and submit a portfolio to the regional approvals panel. A key milestone for a trainee MPAC is achieving MPAC status. Once MPAC status is achieved, an MPAC will move from the trainee role into independently overseeing a clinical caseload across CAMHS inpatient, crisis, and liaison, where they can utilize their expertise to ensure equity of care for patients.

### 4.7 Estimated Caseload for MPAC

Total caseload: 10–15 patients

Inpatient: 4–6 patients (highest intensity)

Crisis: 3–5 patients (medium‐high intensity)

Liaison: 3–4 patients (variable intensity)

#### 4.7.1 Why a Smaller Caseload for Neurodivergent Patients

- Longer appointment times are needed for effective communication
- Sensory accommodations and environmental modifications require more planning
- Complex care co‐ordination with schools, families, and multiple services
- Detailed behavioral support plans and regular reviews
- Higher intensity interventions are often needed
- Communication adaptations such as visual supports

#### 4.7.2 Additional Considerations

- Many neurodivergent individuals have co‐occurring mental health conditions and/or neurodevelopmental conditions
- Family/carer support often requires more intensive involvement
- Transition planning between services can be more complex
- Crisis presentations may be more frequent or intense

The exact number would depend on service resources, staffing levels, and local population needs, but ensuring manageable caseloads is essential for providing individualized, high‐quality care.

### 4.8 Implementation Timeline

#### 4.8.1 Year 1: Pilot Phase

- Recruit 2–3 initial MPACs (or trainees)
- Establish supervision structures
- Develop local protocols
- Begin outcome measures

#### 4.8.2 Year 2: Expansion

- Additional MPAC recruitment
- Peer mentoring program
- Service integration evaluation
- Stakeholder feedback collection

#### 4.8.3 Year 3: Full Implementation

- Complete MPAC team
- Independent credentialling process
- Research and evaluation publication
- Model replication guidance

### 4.9 Integration

Once the MPAC is fully integrated into the team, they will also provide leadership, training, consultation, and advice to CAMHS inpatient, crisis, and liaison and wider services, alongside conducting pioneering research. This role is integral in promoting flexibility within mental health service provision, promoting compassion, care, and inclusion.

### 4.10 Research

- Process evaluation: How well was the model implemented?
- Outcome evaluation: What difference did it make?
- Economic evaluation: Cost‐effectiveness analysis
- Stakeholder views: staff, patient, family perspectives

## 5 Conclusion

The introduction of the MPAC role within CAMHS inpatient, crisis, and liaison represents a strategic investment in the future of CAMHS. This will enhance quality and service efficiency and deliver financial benefits. The MPAC's role will be pivotal in advancing the standard of care provided to neurodivergent populations. In addition, the MPAC role will complement the existing AC role and expand the breadth and depth of crisis‐focused mental health care available by ensuring person‐centered care.

## Author Contributions

The author would like to acknowledge clinical directors, clinicians, and inpatient service managers, as well as regional approvals panel members, for their contribution to informal discussions about the role of an MPAC.

## Ethics Statement

The author has nothing to report.

## Conflicts of Interest

The author declares no conflicts of interest.

## Data Availability

Data sharing is not applicable to this article as no data sets were generated or analyzed during the current study.
